# Complementary Roles of Hippocampus and Medial Entorhinal Cortex in Episodic Memory

**DOI:** 10.1155/2008/258467

**Published:** 2008-07-06

**Authors:** P. A. Lipton, H. Eichenbaum

**Affiliations:** Center for Memory and Brain, Department of Psychology, Boston University, 2 Cummington street, Boston, MA 02215, USA

## Abstract

Spatial mapping and navigation are figured prominently in the extant literature that describes hippocampal function. The medial entorhinal cortex is likewise attracting increasing interest, insofar as evidence accumulates that this area also contributes to spatial information processing. Here, we discuss recent electrophysiological findings that offer an alternate view of hippocampal and medial entorhinal function. These findings suggest complementary contributions of the hippocampus and medial entorhinal cortex in support of episodic memory, wherein hippocampal networks encode sequences of events that compose temporally and spatially extended episodes, whereas medial entorhinal networks disambiguate overlapping episodes by binding sequential events into distinct memories.

## 1. THE BRAIN's GPS

Does hippocampal activity embody the cognitive map? One should expect the neural instantiation of Tolman's [1] cognitive map to contain units (neurons) that are fully allocentric, that is, identify places in the environment independent of the subject's perspective (egocentric direction) and ongoing behavior. Furthermore, one should expect that the neural ensemble composed of these units would be holistic; that is, all the neuronal representations should be tied to one another and change together between environments. And, if the map is to suit the purpose Tolman proposed in guiding behavior according to expectancies, the map should signal the locations of current goals.

Initially, hippocampal place cells seemed to satisfy key criteria for elements of Tolman's cognitive map. The first complete study characterized place cells as signaling an animal's location in the environment independent of egocentric direction and ongoing behavior, as would be expected of the units in an allocentric representation [2]. An expansive literature followed on the initial observations, and many interpreted the results as support for the claim that the neural substrate for the cognitive map lies in the circuitry of the hippocampus [3–9].

However, even in the early data there were loose ends. Location related hippocampal neuronal activity tells us only where the animal is, not where it plans to go, as is Tolman's intended function of a cognitive map [10]. Succeeding studies directly refuted the idea that the hippocampal network contains purely allocentric representations and a holistic map. Inconsistent with a holistic representation, simultaneously recorded place cells respond differently and independently to changes in environmental cues or task demands (e.g., [11–14]). Furthermore, inconsistent with allocentric representation, the activity of most hippocampal neurons is dependent on egocentric spatial parameters, including the direction and speed of the animal's movements [15]. Indeed, place cells reliably provide an allocentric signal only under highly constrained conditions where all perceptual cues, behaviors, and cognitive demands are held constant. In addition, hippocampal neuronal activity has been associated with a variety of nonspatial cues, behaviors, and task demands [16–27], consistent with additional findings showing a critical role for the hippocampus in nonspatial as well as spatial learning and memory [28–30]. Also, several recent studies have provided compelling evidence that so-called place cells are strongly influenced by nonspatial cognitive demands in animals performing spatial memory tasks [31–37], and thus signal where the animal is only in particular circumstances associated with a behaviorally salient task.

In sum, place cells do identify where the animal is when important things happen. But place cells do not carry a reliable allocentric signal, and populations of place cells do not operate as a holistic representation of space or anticipate the locations of goals. Therefore, hippocampal neurons do not have the requisite properties to support Tolman's cognitive map. By contrast, the findings indicate that hippocampal neurons represent events in the places where they occur, consistent with current views of hippocampal involvement in episodic memory (e.g., [38, 39]).

The recent discovery of spatial firing patterns in the cortex immediately adjacent to the hippocampus has refocused the search for the cognitive map to a zone within the medial entorhinal area [36, 40–43]. A majority of the data describes the spatial firing patterns of principal neurons in the medial entorhinal cortex, and more specifically how a proportion of these neurons, the so-called “grid cells,” exhibit an intriguing and unique spatial firing pattern with several interesting properties. First, the relative angles and densities of peaks within grids of neighboring cells remain invariant both across environments and in response to changes in local cues [41]. Second, while grid fields of medial entorhinal neurons remain stable in response to modest environmental manipulation, hippocampal CA3 neurons change their rate of firing (“rate remapping,” [44]). In response to more significant environmental change, grid fields of local ensembles of medial entorhinal neurons rotate while maintaining relative geometric consistency, whereas CA3 neurons fire in a different location (“global remapping”), [44]. Thus, in response to environmental manipulation, changes in medial entorhinal activity are more systematic and predictable than corresponding hippocampal CA3 responses, and consequently more stable as sensory inputs change. Third, while lacking any obvious topographic organization of space, the relative size of medial entorhinal grid fields changes systematically along a dorsal-ventral axis [41]. Although medial entorhinal cells are influenced by egocentric parameters of head direction and velocity, [42], these findings modestly suggest that some version of the cognitive map may reside within the medial entorhinal cortex, rather than the immediately adjacent hippocampus. This interpretation will be argued in several other papers of the current volume. However, here we will suggest an alternate view driven by recent data that includes our own experiment wherein sensory cues were held constant throughout the experiment [36]—that spatial representations observed in medial entorhinal cortex may make a specific contribution to episodic memory.

## 2. EPISODIC MEMORY

### 2.1. Memory for order

The hippocampus is strongly implicated in spatial memory and navigation as evidenced by both behavioral and physiological studies. At the same time, a convergent stream of behavioral, physiological, and computational modeling data indicate that hippocampal processing is critical for episodic memory [45–57]. How can these two seemingly distinct lines of evidence be reconciled?

Current conceptions of episodic memory emphasize the temporal organization of sequences of events as they unfold over time and space [58]. Representations of events are composed as associations between specific objects, actions, and the locations where they occur. Complete episodes are composed of unique sequences of events [38]. Recent experiments have revealed a critical role of the hippocampus in memory for sequences of events that compose unique episodes [47, 59]. In addition, episodic memory also relies on the capacity to distinguish event sequences that share common elements [60]. This property of episodic memory is especially evident in spatial memories, for example, we are usually very good at remembering unique events that occur day by day as we take the same route to work each day. Computational models suggest that the ability to disambiguate overlapping elements from multiple experiences may be a critical feature of hippocampal function that contributes to episodic memory [53]. Consistent with this view, rats with hippocampal lesions fail on a sequence disambiguation task that involved two series of events that contain overlapping items [45].

Additional support for sequencing and disambiguation of serial events by hippocampal networks comes from analyses of hippocampal neuronal activity in animals performing spatial memory tasks. In one study, rats were trained on the classic spatial T-maze alternation task in which successful performance depends on distinguishing left- and right-turn episodes to guide each subsequent choice [37]. If hippocampal neurons encode each sequential behavioral event within one type of episode, then neuronal activity at locations that overlap in left- and right-turn trials should vary according to trial type. Indeed, virtually all cells that were active as the rat traversed these common locations were differentially active on left- versus right-turn trials. Despite modest differences in the proportion of neurons that exhibit this pattern of activity across studies—likely due to differences in training protocols—similar results have been observed in several versions of this task [31–35, 37, 61]. These findings suggest a reconciliation of the spatial and episodic memory views of hippocampal function: place cells represent the series of places where events occur in sequences that compose distinct episodic memories.

### 2.2. Temporal context

In order to correctly trigger a series of event representations within a particular episode, the hippocampus requires a mechanism to bind its representations of event sequences according to the appropriate episode they compose. One suggestion is that sequences are bound by a shared temporal context [49, 62] and that the mechanism for contextual binding involves context sensitive neurons that fire for prolonged periods to bridge sequences of events that occur within a particular context [63]. Here, we review evidence suggesting that the context sensitive neurons exist in the medial entorhinal cortex and serve a function complementary to that of hippocampal place cells which encode discrete events.

Thus far, all observations of grid field activity patterns in medial entorhinal cortex are derived from animals foraging in random directions within an open field. In fact, Derdikman et al. [64] report that the grid structure breaks down when animals are constrained to make hairpin turns within the previously unconstrained open field. This is notable because in the standard, random foraging experimental protocol, spatial cues provide the only regularities and constraints. In contrast, what differed between the hairpin turn maze and the open field condition was the imposition of behavioral constraints; spatial cues were held constant. Importantly, it is only under the unconstrained open field condition that hippocampal cells display purely allocentric spatial firing patterns. Perhaps where stimulus or behavioral regularities are imposed, the activity of neurons in medial entorhinal cortex, like neurons in the hippocampus, might reflect the corresponding regularities embedded in the task protocol.

In a recent study, we adopted the same spatial memory task used previously [37] to compare the activity of hippocampal and medial entorhinal neurons in animals performing a continuous spatial alternation on a T-maze in which hippocampal neurons encode sequences of locations traversed and disambiguate overlapping routes [36]. Two important considerations are worth mentioning here. First, we were explicitly interested in comparing how medial entorhinal and hippocampal neurons uniquely represent aspects of the continuous spatial alternation, rather than in an analysis of grid cell properties. Our interpretation of our data therefore addresses the contribution of medial entorhinal cortex to episodic memory, not whether a grid field forms on a T-maze. Second, just as the expansive place cell literature relies almost exclusively on observations of hippocampal activity in situations that neither require any manner of hippocampal processing nor impose any memory demands, our experimental design exploited the capacity of medial entorhinal neurons to encode spatial information. Whether the task is hippocampal or entorhinal dependent is not relevant to our interpretations. Insofar as the continuous spatial alternation is not a hippocampal dependent-task, it is worth noting that hippocampal dependence is neither an operational definition of, nor a pre-requisite for, memory.

**Figure 1:**
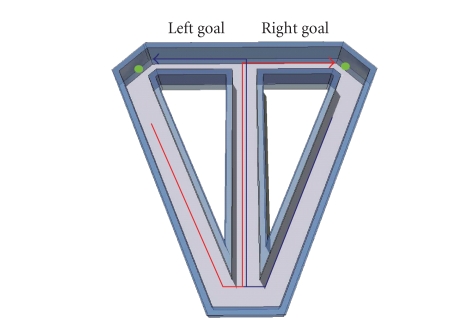
T-maze continuous alternation. Blue line indicates left-turn trial; red line indicates right-turn trial. Small green circles represent reward sites.

**Figure 2:**
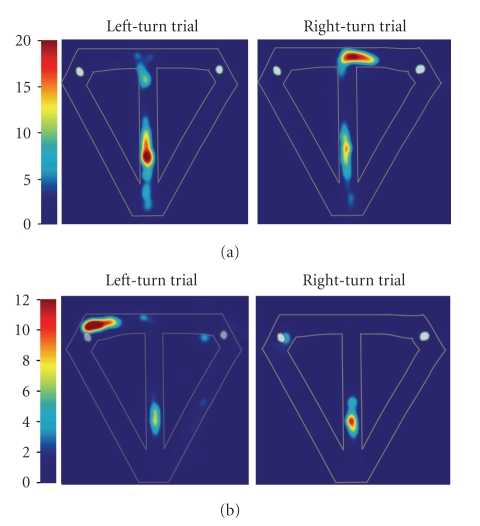
The activity of two example hippocampal neurons represented as false-color rate maps to illustrate differential firing on left- versus right-turn trials. (a). This unit was significantly more active on left-turn trials: significant main effect of segment (*F*_6,420_ = 91.05; *P* < .00001) and interaction (*F*_6,420_ = 2.58; *P* < .02); log-likelihood ratio = 0.2; *p*_correct_ = 0.6; *p*_chance_ = 0.08. (b). This unit was significantly more active on right-turn trials: significant main effect of segment *F*_6,252_ = 68.3; *P* < .00001) and interaction *F*_6,252_ = 2.92; *P* = 0.009); log-likelihood ratio = 1.22; *p*_correct_ = 0.63; *p*_chance_ = 0.07. Color bars indicate firing rate in Hz.

**Figure 3:**
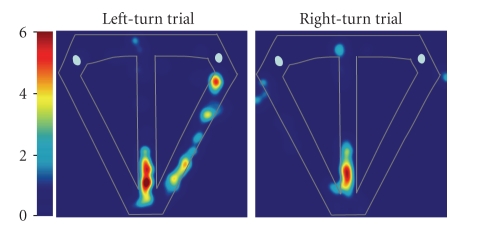
Location related firing of a medial entorhinal neuron that did not fire differentially on left- versus right-turn trials. Significant main effect of segment (*F*_6,238_ = 44.43; *P* < .00001); loglikelihood ratio = 2.5; *p*_correct_ = 0.72; *p*_chance_ = 0.006. Color bars indicate firing rate in Hz.

We trained rats to perform the spatial alternation task on a T-maze that included return arms that connected the end of each goal arm to the starting end of the central stem (Figure 1). A left-turn trial began as the animal departed the right goal area, ran down the return arm to the central stem, traversed the central stem, and made a left-turn into the left goal area to retrieve a water reward. Similarly, a right-turn trial began when the animal departed the left goal area, returned to and traversed the central stem, and made a right turn into the right goal area. Drawing on the model of episodic memory noted above, each left- or right-turn trial can be considered a unique episode, constructed by connecting sequential behavioral events identified by a series of loci along the maze. Areas that lie along the central stem constitute overlapping elements of both types of episodes, and are indeed represented differently by hippocampal neurons depending on the ongoing episode (Figure 2, [36, 37]). Furthermore, consistent with previous reports [40, 65], activity of neurons in medial entorhinal cortex also signals an animal's position along the maze. Though we did not witness the development of a grid-like firing pattern on the T-maze, a proportion of our medial entorhinal neurons did exhibit a high degree of spatial specificity [36]. For example, the medial entorhinal cell shown in Figure 3 fired predominantly at the proximal end of the central stem during both left- and right-turn trials, while remaining largely silent through other regions of the maze.

**Figure 4:**
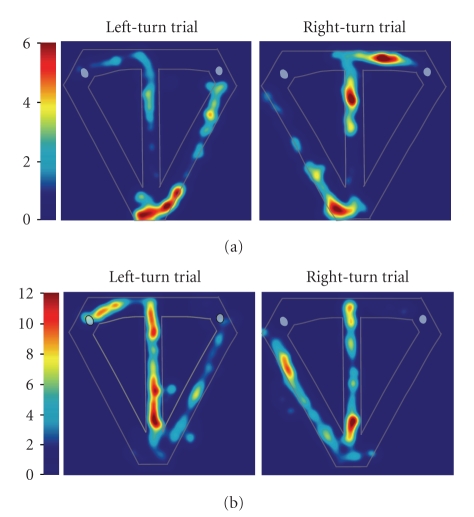
Firing patterns of two representative medial entorhinal neurons that reflect both trial disambiguation and a low degree of spatial specificity. (a). This unit was significantly more active on right-turn trials: significant main effect of segment (*F*_6,392_ = 11.73; *P* < .00001), trial type (*F*_1,392_ = 7.32; *P* < .00071) and interaction (*F*_6,392_ = 2.22; *P* < .04); log-likelihood ratio = 4.62; *p*_correct_ = 0.67; *p*_chance_ = 0.006. (b). This unit was significantly more active on left-turn trials: significant main effect of segment (*F*_6,280_ = 3.83; *P* < .0011), trial type (*F*_1,280_ = 4.87; *P* < .03) and interaction (*F*_6,280_ = 1.83; *P* < .09); log-likelihood ratio = 1.29; *p*_correct_ = 0.71; *p*_chance_ = 0.004. Color bars indicate firing rate in Hz.

Many of our medial entorhinal neurons that exhibited spatial specificity also exhibited differential firing along the central stem of the maze during left- and right-turn trials, similar to hippocampal neurons [36]. The patterns of neuronal activity illustrated in Figure 4 represent typical trial-type specific activity exhibited by medial entorhinal neurons. Some medial entorhinal cells fired selectively during the trial and distinguished left-turn and right-turn trials. For example, the cell shown in Figure 4(a) was selectively active when the rat was near the end of the central stem and fired at a higher rate during right-turn compared to left-turn trials. However, most medial entorhinal neurons showed only crude spatial specificity. For example, the cell shown in Figure 4(b) fired somewhat indiscriminately through different regions of the maze, and although active along the entire central stem, was significantly more active on left-turn trials. This pattern of activity was an exclusive feature of medial entorhinal neurons, such that we observed no hippocampal units with poorly localized, trial-type specific firing that extended the length of the central stem [36].

We used a two-way ANOVA and log-likelihood estimation to quantitatively compare the incidence and robustness of trial-type disambiguation in medial entorhinal and hippocampal neurons. Dividing the central stem into seven equal segments, we used a two-way ANOVA to compare the spatial firing patterns on segments of the central stem between left-turn and right-turn trial types for each cell [37]. We considered that a significant main effect of trial type or a trial type by segment interaction qualified a cell as differentiating left- from right-turn trials. A significant main effect of segment without a significant main effect of trial type or trial type by segment interaction denoted location-specific activity only. The log-likelihood ratio [66], on the other hand, represented the degree to which firing patterns on left-turn and right-turn trials differed, and thus allowed us to measure the difference in the firing patterns across trial types, rather than knowing simply that they differed. The log-likelihood ratio was calculated as follows: 1*n* *p*[*r* | *L*, *x*]/*p*[*r* | *R*, *x*], where *p*[*r* | *L*, *x*] is the probability density function of left-turn (*L*) trials at position *x*, evaluated at the observed firing rate *r*, and *p*[*r* | *R*, *x*] is the equivalent function for right-turn (*R*) trials [66]. For each cell, log-likelihood ratios were summed over all central stem bins for each trial. Where the log-likelihood sum is greater than zero, maximum likelihood analysis predicts that the data came from a left-turn trial; otherwise, a right-turn trial is predicted. We calculated the average absolute value of the summed log-likelihood ratio, such that larger values of this term indicate firing-rate patterns that are statistically more distinct (for a more detailed description, see [36]).

Using the two-way ANOVA, we identified neurons in the hippocampus and medial entorhinal area that distinguished trial type as animals traversed the central stem. Based on the two-way ANOVA, 56% of medial entorhinal neurons (23/41 with place fields on the central stem) were significantly more active on either right- or left-turn trials, whereas 33% (16/48) of hippocampal neurons exhibited differential firing on the central stem. Moreover, the log-likelihood estimation revealed that medial entorhinal neurons more robustly distinguished left- from right-turn trials than did hippocampal neurons, such that the average log-likelihood ratio for medial entorhinal neurons was significantly greater than for hippocampal neurons (MEC, 2.82; Hippocampus, 1.7; Wilcoxon rank-sum test, *P* < .003). In other words, firing patterns of medial entorhinal neurons were on average more distinct on either left- or right-turn trials than were hippocampal neurons.

Two additional measures were applied to describe how the patterns of activity in hippocampal and medial entorhinal neurons differed along the central stem during left- and right-turn trials [36]. The first measure, *p*_correct_, is based on a maximum-likelihood guess performed for each trial compared against the actual outcome of that trial, and thus describes how accurate the log-likelihood estimate is for each trial type. To calculate *p*_correct_, we performed a maximum likelihood analysis using the conditional density functions as described above. *p*_correct_ represents the number of times that prediction was correct, divided by the total number of trials. Therefore, *p*_correct_ is the average trial-by-trial probability that the log-likelihood analysis gives the correct answer for each trial type: a population that more consistently and significantly differentiates left- from right-turn trials will have a higher *p*_correct_. The second measure, *p*_chance_, is the average probability that firing patterns across left- and right-turn trials arose by chance, given a *p*_correct_ of 0.5 (i.e., the firing rate contains no trial-specific information, which is necessary to avoid biasing the calculation). To calculate *p*_chance_, we evaluated the following formula: Pnk = *n*!/*k*!/(*n* − *k*)!(0.5^*k*^)0.5^(1−*k*)^, where *n* is the number of trials, and *k* is the number of apparently correct answers from maximal likelihood analysis. To get *p*_chance_, we summed Pnk for all values of *k* greater than or equal to the number associated with our measured value of *p*_correct_. A *p*_chance_ = 0.05 determined that a cell could successfully distinguish trial type.

Again, medial entorhinal neurons had a significantly higher mean *p*_correct_ than hippocampal neurons (MEC, 70%; Hippocampus, 63%; Wilcoxon rank-sum test, *P* < .0001), indicating that the activity of medial entorhinal neurons along the central stem more successfully predicted trial type than hippocampal neurons. Correspondingly, the difference in firing among left- and right-turn trials of medial entorhinal neurons was on average less likely to have occurred by chance than that of hippocampal neurons (*p*_chance_ equal to or less than 0.05: 90% MEC; 50% Hippocampus).

While the ANOVA and log-likelihood estimation did not always agree on specific units, together they converged on the same conclusion; just as hippocampal units did not exclusively encode information about space, medial entorhinal neurons likewise exhibited location-related firing modulated by mnemonic demands. Furthermore, medial entorhinal neurons performed better than hippocampal neurons at distinguishing trial type on our version of the continuous spatial alternation [36].

Conversely, hippocampal neurons showed greater spatial specificity than medial entorhinal neurons, as is evident by directly comparing the spatial firing patterns displayed in Figures 3 and 4. Our visual observations were bolstered by three quantitative measures—performed on all hippocampal and medial entorhinal units with a firing field somewhere on the maze—meant to assess spatial selectivity: place field size, spatial tuning, and spatial information rate. On all three measures hippocampal and medial entorhinal activity differed significantly. For example, average hippocampal place field size for all units with location related activity on the maze was significantly smaller than that of medial entorhinal neurons (256.8 cm^2^ versus 330.8 cm^2^, resp.; Wilcoxon rank-sum test, *P* < .0003). The degree of spatial tuning, or the ratio of firing inside versus outside a place field, for hippocampal neurons was on average significantly higher than for medial entorhinal neurons (11.5 versus 3.0, resp.; Wilcoxon rank-sum test, *P* < 8.8E–16). The amount of spatial information conveyed by hippocampal neurons also was significantly greater than that of medial entorhinal neurons (2.02 bits/second versus 0.89 bits/second, resp.; Wilcoxon rank-sum test, *P* < .00001).

Together the results of this study suggest that disambiguation of overlapping experiences occurs prior to the hippocampus, and that hippocampal and medial entorhinal circuits play distinct and complementary roles in the continuous spatial alternation. Medial entorhinal neurons more successfully distinguished task related episodes in the context of left- versus right-turn trial type, whereas hippocampal neurons provided a greater degree of spatial specificity. Together both regions supply requisite elements of a neural code for particular events as they occur within unique episodes.

Since the neural circuitry among these brain regions constitutes a series of loops [67], it is difficult to positively attribute specific functions to individual brain regions. However, very recent evidence from observations of CA1 neuronal activity in animals with lesions to layer III of medial entorhinal cortex offers crucial support [68]. The results demonstrate that precise spatial coding of CA1 neurons is more dependent on this direct entorhinal input than on projections from CA3 which provide indirect input from layer II of medial entorhinal cortex [68], indicating that the manner of spatial information processing most commonly observed in CA1 is the result of a clear progression from medial entorhinal cortex to hippocampus.

## 3. HOW DOES THE MEDIAL ENTORHINAL CORTEX CONTRIBUTE TO EPISODIC MEMORY?

Our recent experimental results confirm that medial entorhinal neurons carry a spatial signal. However, as noted above, most of these neurons do not fire at discrete locations associated with particular trial events, as do hippocampal neurons. Instead, many medial entorhinal cells show strong context sensitivity, outperforming hippocampal neurons in distinguishing left-turn and right-turn trials. Furthermore, the prolonged firing periods of medial entorhinal cells are consistent with the characterization of context sensitive neurons that could bind a series of hippocampal representations of punctate events [63].

A growing body of evidence supports the notion that the medial entorhinal area is part of the parahippocampal region that processes contextual representations. This evidence is derived from knowledge about the anatomical pathways of the hippocampal system and from recent functional imaging studies [39]. Inputs to the hippocampus arrive via the surrounding cortical areas that compose the parahippocampal region [67]. This region can be subdivided into the perirhinal cortex, the parahippocampal cortex (called postrhinal cortex in rodents), and the entorhinal cortex. Most neocortical inputs to the perirhinal cortex are derived from association areas that process unimodal sensory information about qualities of objects (i.e., “what” information), whereas most of the neocortical inputs to the parahippocampal cortex (called postrhinal cortex in rats) originate in areas that process polymodal spatial (where) information.

Subsequently, the “what” and “where” streams of processing remain largely segregated as the perirhinal cortex projects primarily to the lateral entorhinal area, whereas the parahippocampal cortex projects mainly to the medial entorhinal area. While there are also some connections between the perirhinal and parahippocampal cortices and between the entorhinal areas, the “what” and “where” information mainly converge within the hippocampus. Hippocampal efferents back to the cortex involve feedback connections from the hippocampus successively back to the parahippocampal region and thence to neocortical areas from which the inputs originated. This anatomical evidence suggests that, during encoding, “what” information carried in the perirhinal-lateral entorhinal stream is combined with “where” information carried in the parahippocampal-medial entorhinal stream and the hippocampus associates items and their spatial context. When an item is subsequently presented as a memory cue, the hippocampus completes the full pattern and mediates a recovery of the contextual representation in the parahippocampal cortex and medial entorhinal area, and the recovery of context constitutes the experience of episodic recollection.

In support of this model, evidence from functional imaging studies in humans indicates that the parahippocampal cortex component of the “where” stream represents spatial context. One line of evidence comes from the work of Kanwisher and colleagues, showing that the parahippocampal region is activated when people view spatial scenes and not objects or faces [69]. The other line of evidence comes from work of Bar and colleagues, showing that the parahippocampal cortex is activated when people view objects that have strong spatial contextual associations (e.g., a refrigerator, a roulette wheel, [70]). Similarly, a cellular (fos) imaging study indicates that the postrhinal cortex also is activated in rats by novel spatial arrangements of cues [71]. In addition, Aminoff et al. [72] reported that adjacent components of the parahippocampal cortex are activated by spatial context, and that this activity emerges as people view abstract patterns that were elements of newly learned spatial patterns or simply temporally associated. These findings extend the potential role of the parahippocampal cortex to temporal contextual representations as well as spatial context. Such a view is consistent with the frequent observation that the parahippocampal region is activated when humans recollect items in the context in which they were learned (reviewed in [39]).

We lack studies that compare response properties of the parahippocampal cortex and the medial entorhinal area. However, the combined data from functional imaging of the parahippocampal cortex in humans and animals and our recent study of spatial firing properties of medial entorhinal neurons suggest that both the parahippocampal cortex and medial entorhinal area components of the “where” pathway may be specialized for the processing of spatial and temporal context in humans and animals. Much work remains to be done to test this hypothesis. However, we believe there is sufficient evidence to consider the medial entorhinal area as part of a contextual representation system rather than the embodiment of a cognitive map that guides spatial navigation.
